# Prevalence of occupational injuries and associated factors among solid waste collectors in Jigjiga city, eastern Ethiopia: a cross-sectional study design

**DOI:** 10.3389/fepid.2024.1439038

**Published:** 2024-11-12

**Authors:** Muktar Abib, Sina Temesgen Tolera, Abdiwahab Hashi, Yohannes Mulugeta, Liku Muche Temesgen

**Affiliations:** ^1^ Smart Consulting Firm, Jigjiga, Ethiopia; ^2^ Department of Environmental Health, College of Health and Medical Sciences, Haramaya University, Harar, Ethiopia; ^3^ Department of Public Health, Jigjiga University, Jigjiga, Ethiopia

**Keywords:** accidents, occupational injuries, solid waste collectors, Jigjiga city, Ethiopia

## Abstract

**Background:**

Solid waste collectors play an important role in maintaining health and hygiene in cities globally. The risk levels are very high in low-income countries since solid waste collectors have low socio-economic status and are exposed directly, unknowingly, and without adequate personal protection to municipal solid waste that contains hazardous materials. Solid waste collectors in Ethiopia are at high risk of occupational injuries due to the manual collection of hazardous solid waste by hand. In Jigjiga city, so far, there has been no study or published research showing the prevalence of occupational injuries and associated factors among municipal solid waste collectors.

**Objective:**

To assess the prevalence of occupational injuries and associated factors among solid waste collectors in Jigjiga City, Somali Regional State, Ethiopia, 2023.

**Methods:**

An institutional cross-sectional study was conducted in Jigjiga City, Somali Regional State, involving 247 solid waste collectors. Data was collected through an observational and structured questionnaire and analyzed using Epi Info and STATA software programs. The study used systematic random sampling techniques and bivariate and multivariable logistic regression analyses to determine the statistical association between the outcome variable and independent variables. The significance of the association was tested using the 95% confidence interval and *p* value (<0.05).

**Results:**

The overall prevalence of occupational injuries was 54.7% (95% CI: 48.2%, 60.6%). Training on health and safety measures before employment [AOR: 0.43, 95% CI (0.24, 0.80)], sleeping problems [AOR: 3.28, 95% CI (1.86, 5.78)] and Temporary workers [AOR: 2.14, 95% CI (1.16, 3.95)] were significantly associated with occupational injuries.

**Conclusion:**

The prevalence rate of occupational injuries among solid waste collectors in Jigjiga City was high. There should be preventive measures, like giving Training on Health and safety before employment, to safeguard the health and safety conditions of the workers.

## 1 Introduction

Solid waste, including garbage, refuse, and discarded materials, is produced annually in various categories like municipal solid waste (MSW), healthcare waste, and e-waste. With over 2 billion tons produced, the sector is the sixth most dangerous occupation globally, with high rates of work-related injuries. MSW collectors face injuries at every stage of waste management (1, 2). Globally, there are 2.3 million deaths each year that are related to the Solid waste workplaces. The largest portion, 2.0 million, is related to occupational diseases, and 0.3 million to occupational injuries (3). The world's manual waste collection is one of the riskiest jobs, involving physical, chemical, and biological exposures, as well as stress and inadequate safety precautions. Common issues include injuries, respiratory and musculoskeletal disorders, slips, and traffic accidents. Waste collectors, particularly those in their middle years and those with less education, face significant health risks (4–6).

Regardless of the degree of development of a nation, solid waste collectors are vulnerable to occupational health problems. However, the level of risk faced by solid waste collectors (SWC) varies greatly globally (7). The World Health Organization (WHO) estimates that occupational diseases account for around 11% of the global disease burden (8). Developed countries have reduced occupational and environmental hazards from municipal solid waste through proper waste management, but low-income countries still face high risks for MSW collection workers due to low socioeconomic status and lack of adequate personal protective equipment (9). Despite advances in waste management practices, a recent study found that large numbers of workers across all economic sectors are still being injured on the job (10).

Furthermore, work-related accidents and injuries pose a significant risk to both developed and developing nations, causing significant financial and human costs. Current data links 3,400,000 incapacitating injuries to work-related events, with fatal injuries occurring every two hours and disabilities every eight hours (11, 12). The risk of occupational injury is particularly high in developing countries, especially in Africa. A study conducted in sub-Saharan Africa found that solid waste collectors are exposed to many risks while collecting waste, such as bad odors, sharp materials, dust, and harsh flies (13). Injuries at work are unplanned and unforeseen, and they can result in fatal or non-fatal injuries to at least one body part (14). As the studies reveals that the highest rate of occupational injuries appears to be in Sub-Saharan African nations (15, 16).

On the other hand, Occupational diseases and injuries cost an average of 1.8%–6.0% of a nation's Gross domestic product (GDP), negatively impacting the economy, particularly in emerging countries, with sub-Saharan African nations having the highest rate of occupational injuries (3). Sub-Saharan African nations suffer from the highest prevalence of work-related injuries, with over 42 million work-related accidents annually (17). The risk of occupational injuries among municipal solid waste workers in low- and middle-income countries is increasing, with a study in Ethiopia, Ghana and Uganda revealing a link between the use of personal protective equipment (PPE) and injuries among casual waste workers. In Ethiopia, manual tasks like lifting, carrying, pulling, and pushing are common in the collection of solid waste, with work-related injuries being two to five times higher than in developed countries (18–21).

The high prevalence of occupational injuries among Ethiopian solid waste collectors underscores the need for comprehensive interventions to address health and safety concerns. Poor waste handling practices exacerbate these injuries, making the solid waste management sector riskier. The lack of workplace-related studies is concerning (20–24).

The high rate of occupational injuries among solid waste collectors in Ethiopian cities like Addis Ababa, Harar, and the Amhara region is a significant concern. However, there is a lack of research in Jigjiga City, which relies on manual waste collection. Conducting a study in Jigjiga City is crucial to understand the scope and determinants of these injuries, and develop effective interventions to minimize the risks faced by these workers. This research is urgently needed to address this public health issue and ensure the well-being of these workers.

## 2 Methods

### 2.1 Study area and period

**Figure 1:**
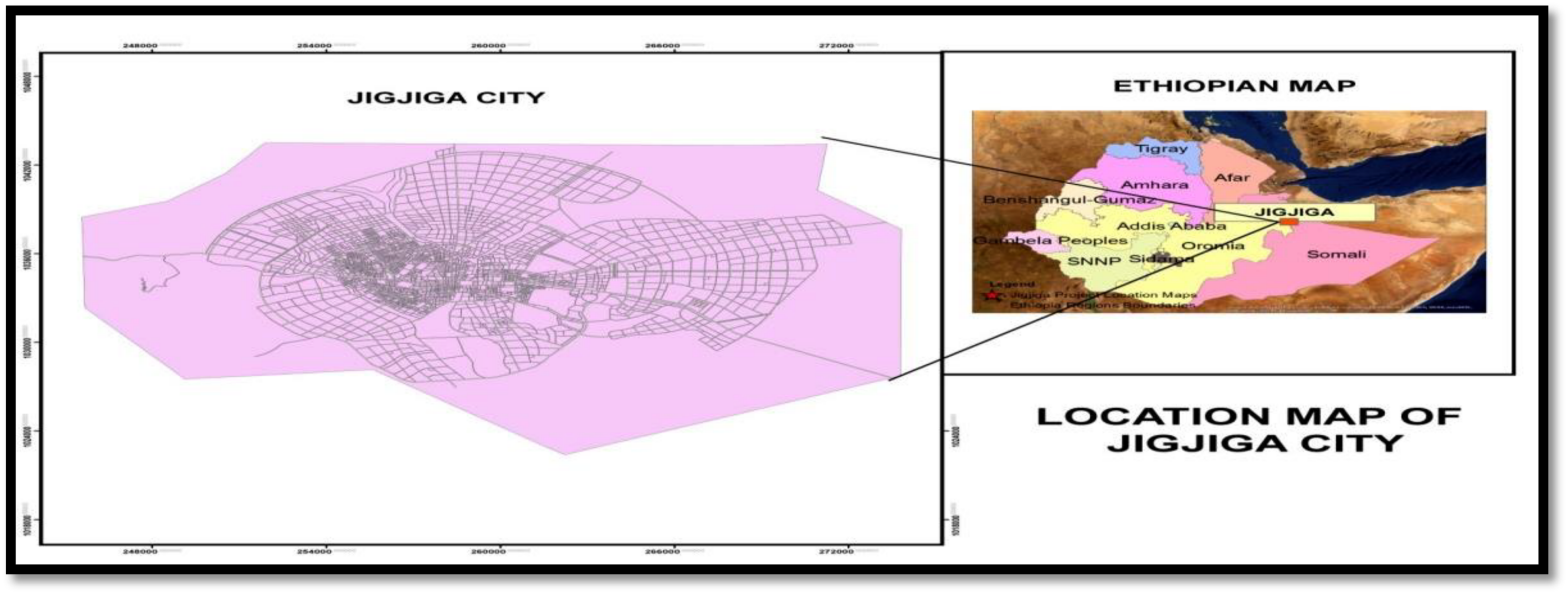
Location Map of Jigjiga city in Somalia region eastern Ethiopia.

Jigjiga, the capital of Somali Regional State, is located in the eastern part of the country, approximately 630 km east of Addis Ababa City. With an elevation of 1,609 m, the city is situated on flat land with gentle slopes, making it ideal for urban development. A study was conducted from June 25th to July 25th, 2023, in Jigjiga, Somalia, to assess the city's sanitation and beautification efforts. The city employs 247 solid waste collectors to serve the 20 Kebeles within the city (Figure 1).

### 2.2 Study design

An Institutional cross-sectional study was conducted to assess occupational injuries and associated factors among Solid waste collectors in Jigjiga City.

### 2.3 Source and study population

All solid waste collectors found in Jigjiga City was the source population. While, All selected solid waste collectors within the kebeles of Jigjiga City were the study population.

### 2.4 Inclusion and exclusion criteria

The study includes all solid waste collectors of both sexes who have been part of the office or enterprise for over a year and agree to participate, except for those with serious illnesses, non-responsiveness, maternity leave, and annual leave.

### 2.5 Sample size determination

The study focuses on the 247 solid waste collectors in Jigjiga City, engaged in both the sanitation and beautification offices and micro and small enterprises. A systematic random sampling technique was employed to select participants (K = 1). The first collector was randomly selected, followed by others who were selected based on the allocated sample size. This comprehensive approach ensures a representative sample of the entire population, ensuring a comprehensive understanding of the waste collection process (Supplementary File Figure S1).

### 2.6 Data collection method and procedure

The study involved solid waste collectors in Jigjiga City who had direct contact with waste. The study utilized an observational and structured questionnaire, prepared in English, translated to Somali and Amharic, and then back to English, to gather data from participants. Four trained diploma holders collected data, with two BSc degree supervisors supervising. The principal Investigator (PI) provided overall supervision for smooth data collection. Data was collected through face-to-face interviews using a pre-tested standard questionnaire from June 25th to July 25th, 2023. Participants provided verbal consent, and the questionnaires were collected and checked daily for consistency and completeness. The pre-tested standard questionnaire was developed based on related published studies with modifications. A pretest was conducted on the nearby city administration for validation. The questionnaire was adapted from relevant literature and used for the study's objectives.

### 2.7 Variables

#### 2.7.1 Dependent variable

Occupational injuries.

#### 2.7.2 Independent variables

• Socio-demographic Factors: Working experience, age of workers, Sex of workers, educational status, marital status, Family size, and Monthly salary.
• Occupational Safety Factors: Work related issues, type of work and working days, Health safety training and education, Availability of PPE & Utilization of PPE.
• Behavioral factors: Use of substances like (chat chewing, alcohol drinking, Cigarette smoking), sleeping problem, job stressfulness.

### 2.8 Data quality control

The possibility of biased information was minimized by translating the English-prepared questionnaire into Somali, making it easier for interviewers and interviewees to understand and administer, and then back to English to ensure consistency. The study used a structured and pre-tested 5% questionnaire to ensure data quality. The questionnaire was amended after pre-testing, and data collectors and supervisors received two days of training. Supervisors frequently checked the information, and the principal investigator provided overall supervision. The questionnaire was checked for completeness every night during data collection, and feedback on previous activities was given to both data collectors and supervisors. Data entry was done twice to reduce errors.

### 2.9 Data analysis methods

The data was visually checked for completeness and coded using Epi Data. Randomly selected responses were checked for consistency and transferred to STATA for analysis. Descriptive statistics were used to provide a coherent presentation, and binary logistic regression was used to identify associations between independent variables and occupational injury. Bi-variable binary logistic regression was used to evaluate the association between predictor variables and dependent variables. Variables with a *p* value < 0.25 were considered for multivariable binary logistic regression analysis. Model fitness was checked using Hosmer–Lemeshow, and multi-collinearity among predictor variables was checked using the variance inflation factor. The significance association was declared at *p*-value < 0.05. Findings were presented with odds and a 95% confidence interval.

## 3 Results

### 3.1 Socio-demographic factors

The data was collected from 246 waste collectors out of the 247 total solid waste collectors, of whom two were incomplete. This study includes 246 solid waste collectors with a responsive rate of 99.6%. The majority of the surveyed participants were 153 (62.45%) males. The mean ages of respondents were 28 (SD ± 9.5). The average family size of the respondents was 4.5, (SD ± 2.5). About 174 (71.02%) were married, 86 (35.1%) were illiterate, and 245 (99.59%) did not have any other job. On the other hand, the average income of the respondents was 3,902.041birr (SD ± 2,147.8) as displayed in Table 1.

**Table 1:** Socio-demographic factors of municipal solid waste collectors in Jigjiga city, eastern Ethiopia, June 25th to July 25th 2023GC (*n* = 246).

Variables		Frequency (*n*)	Percentage%
Sex	Female	92	37.55
	Male	153	62.45
Marital status	Married	175	71.14
	Single	66	26.83
	Divorced/separated	4	1.63
	Widowed	1	0.41
Age	≤18	23	9.35
	19–64	221	89.84
	+65	2	0.81
Educational level	Illiterate	86	34.96
	Can read and write	10	4.07
	Grade 1–4	41	16.67
	Grade 5–8	75	30.49
	Grade 9–12	30	12.20
	Grade 12+	4	1.63
Employment condition	Permanent	140	56.91
	Temporary/contract	106	43.09
Additional Job availability	No	245	99.59
	Yes	1	0.41

### 3.2 Behavioral factors

Regarding behavioral characteristics, 96 (39.02%) of the individuals smoked cigarettes, 124 (50.41%) chewed khat, and 21 (8.54%) had a history of alcohol consumption. However, only 6 (28.57%), 38 (30.65%), and 24 (24.74%) had started drinking alcohol, chewing khat, and smoking after starting employment, respectively. This study also showed that 129 (52.44%) of respondents had sleeping problems and from those 124 (96.12%) were developed sleeping problems after engaged in this job. The workers had work related violence and disagreement 59 (23.98%) and they mostly face disagreement or violence with their manager 26 (44.07%). The data also shows that the majority of the Solid waste collectors 211 (85.77%) are satisfied with this work (Table 2).

**Table 2:** Behavioral characteristics of solid waste collectors in Jigjiga city, eastern Ethiopia, June 25th to July 25th 2023GC (*n* = 246).

Variables	Predictors	Frequency (*n* = 246)	Percentage (%)
Work-related violence and disagreement	No	187	76.02
	Yes	59	23.98
Work-related violence with the	Manager	26	44.07
	Residents	22	37.29
	Colleagues	11	18.64
Smoking cigarettes	No	150	60.98
	Yes	96	39.02
Drinking alcohol	No	225	91.46
	Yes	21	8.54
Chewing chat	No	122	49.59
	Yes	124	50.41
Sleeping problem	No	117	47.56
	Yes	129	52.44
Work satisfaction	No	35	14.23
	Yes	211	85.77
Started smoking cigarettes	Before work	72	75.00
	After work	24	25.00
Started srinking alcohol	Before work	15	71.43
	After work	6	28.57
Started chewing chat	Before work	86	69.35
	After work	38	30.65
Developed sleeping problem	Before engagement	5	3.88
	After engagement	124	96.12

### 3.3 Occupational safety factors

Among respondents, only 64 (26.02%) had personal protective equipment (PPE). Out of those, 50 (76.92%) use PPE while on duty, and 59 (92.19%) are supplied by the cooperative unions. The majority of the respondents, 95 (38.78%), don't get the required safety training from their organization, 129 (52.44%) get on-the-job training on safety issues, and only 62 (25.51%) of the waste collectors get vaccinated with tetanus. The mean working experience, working days, and working hours of the respondents were 4.45, 6.85, and 9.11 with SD ± 6.01, SD ± 0.56, and SD ± 1.63, respectively (Table 3).

**Table 3:** Occupational safety factors of solid waste collectors in Jigjiga city, eastern Ethiopia, June 25th to July 25th 2023GC (*n* = 246).

Variables	Predicators	Frequency (*n*)	Percent (%)
Availability of PPE	No	182	73.98
	Yes	64	26.02
Supplier PPE	From municipality	5	7.81
	From union	59	92.19
Types of PPE available	Glove	1	1.54
	Facemask	3	4.62
	Overall	59	90.77
	Other (specify)	2	3.08
PPE utilization on duty	No	15	23.08
	Yes	50	76.92
Type of PPE in use	Glove	4	8.33
	Facemask	3	6.25
	Boot	1	2.08
	Overall	31	64.58
	Other (specify)	9	18.75
Reasons not to use PPE	Not comfortable to use	5	55.56
	To save time	2	22.22
	Not known reason	2	22.22
Training on Health and safety before employment	No	150	60.98
	Yes	96	39.02
On job Training	No	117	47.56
	Yes	129	52.44
Training given by	From union	82	64.06
	From municipality	40	31.25
	From NGOs	6	4.69
Vaccination for tetanus	No	181	74.18
	Yes	63	25.82
Vaccinated	From your union	59	93.65
	From municipality	4	6.35

### 3.4 Prevalence of occupational injuries

The overall prevalence of occupational injuries was 54.7% (95% CI: 48.2%, 60.6%) in the last twelve months. Of the 134 solid waste collectors injured in the last year, 46 (43.4%) sustained injuries two times, while 33 (31.13%) sustained more than twice. Moreover, 107 (74.83%) workers had injuries in the last month before the data collection, illustrated in the subsequently (Table 4).

**Table 4:** Distribution of occupational injury and injured body parts in the last one year among solid waste collectors in Jigjiga city, eastern Ethiopia, June 25th to July 25th 2023GC (*n* = 246).

Variable	Predictor	Frequency	Percent (%)
Occupational injury in the past 12 months	No	112	45.53
	Yes	134	54.47
Occupational injuries in the last month	No	139	56.5
	Yes	107	43.5
Frequency of injuries	One time	27	25.47
	Two times	46	43.40
	more than twice	33	31.13
What you do at Injury time	Collecting waste	95	73.08
	Lifting waste	20	15.38
	Loading cart	11	8.46
	Unload cart	1	0.77
	Loading container	1	0.77
	Others	2	1.54
No of days out of the work due injury	≤3 days	72	55.38
	>3 days	58	44.62
Treatment received	No	82	63.08
	Yes	48	36.92
Where treated	Health facility	37	72.55
	Home	12	23.53
	Herbalist	2	3.92

**Figure 2:**
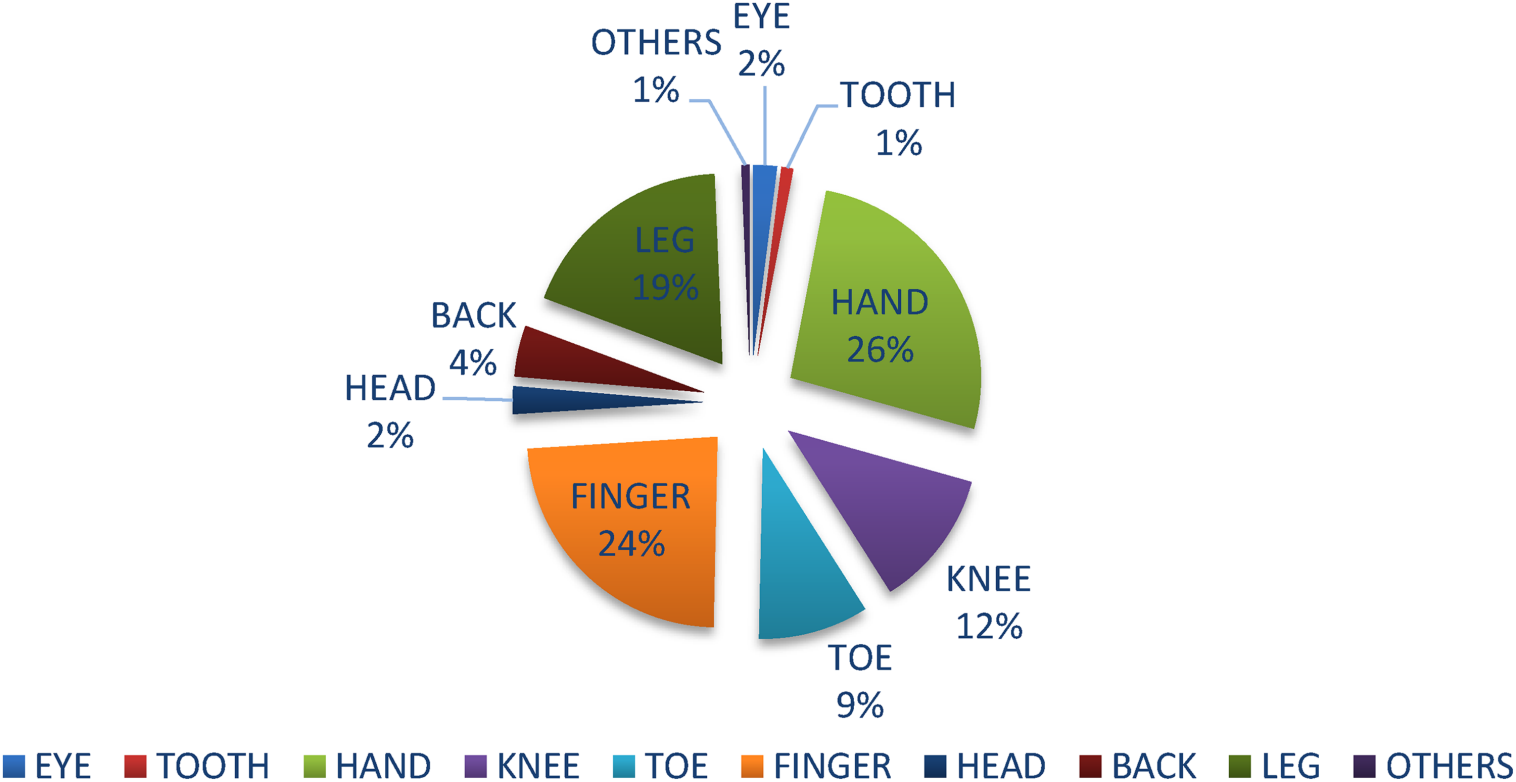
Affected body parts of solid waste collectors in Jigjiga city, eastern Ethiopia.

Regarding the injured body parts of the respondents, 230 (60.4%) has experienced injuries. The most common injured body parts were 79 (26.49%) hand injuries, followed by 71 (24%) finger injuries, 56 (19%) legs, 35 (12%) knee, and 28 (9%) toes and the least were others (Neck, shoulder, Tendon and muscle strains, Ligament Sprains and Tears) (Figure 2).

**Figure 3:**
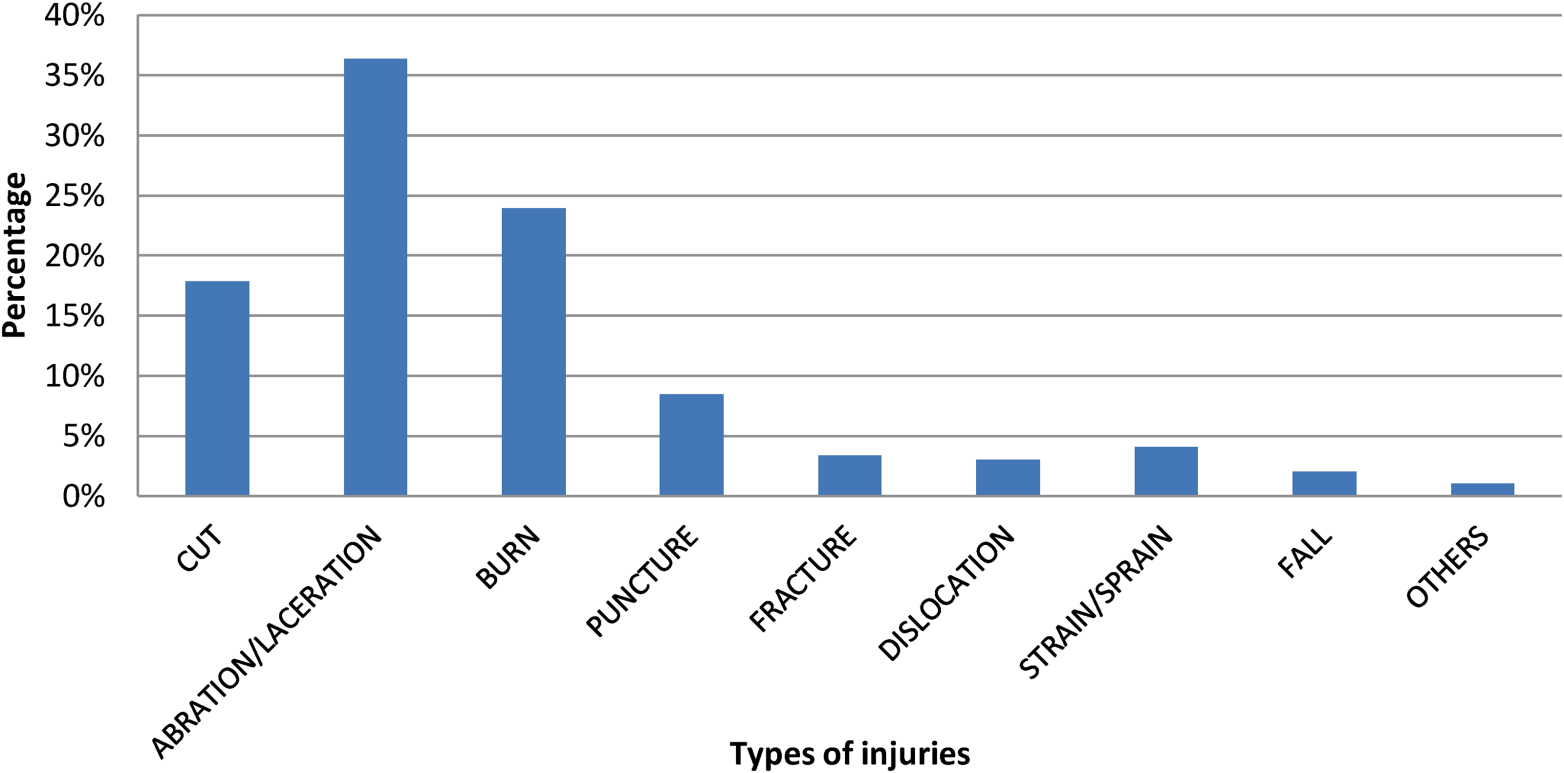
Types of occupational injuries of solid waste collectors in Jigjiga city, eastern Ethiopia.

The most common types of occupational injuries among solid waste collectors were 108 (36%) aberrations or lacerations, 71 (24%) burns, 53 (18%) cuts, and 25 (8%) punctures. 72 (55.38%) of the 134 respondents who have been injured in the last twelve months have missed one to three workdays, while 58 (44.62%) missed more than three working days. Most of the workers have faced injuries while collecting the waste, 95 (73.08%), and only 48 (36.92%) of them have received treatment, with 37 (72.55%) receiving treatment at a health facility (Figure 3).

### 3.5 Factors associated with occupational injuries

#### 3.5.1 Bivariate logistic regression analysis

In the bivariate model, occupational injuries were significantly associated with Training on Health and safety before employment, Work related violence, Sleeping problem, job satisfaction and Employment Condition. In a bivariate logistic regression model, variables such as sex, age, marital status, Educational level, work experience, working days, monthly salary, PPE use on duty, on-job training, smoking cigarette, drinking alcohol and chewing chat were not associated to occupational injuries. Variables such as Training on Health and safety before employment, Work related violence, sleeping problem, job satisfaction and Employment Condition were chosen for the final model to control the effect of confounders (Table 5).

**Table 5:** Bivariate logistic regression of occupational injury with predictor variables among solid waste collectors in Jigjiga city, eastern Ethiopia, June 25th to July 25th 2023GC (*n* = 246).

Variables	Predictor	Occupational injuries in the past 12 months (246)		Crude odds ratio (95% CI)
		Yes	No	
Sex	Female	46	47	1.00
	Male	88	65	1.38 (0.82, 2.32)
Age (year)	≤18	14	9	1.00
	19–64	119	102	0.75 (0.31, 1.80)
	65+	1	1	0.64 (0.035, 11.6)
Educational level	Illiterate	39	47	2.4 (0.249, 24.895)
	Can read and write	5	5	2.99 (0. 23, 39.47)
	Grade (1–4)	27	14	5.78 (1.07, 60.53)
	Grade (5–8)	45	30	4.50 (0.45, 45.38)
	Grade (9–12)	17	13	3.92 (0.36, 42.20)
	Grade 12+	1	3	1.00
Marital status	Married	75	100	1.00
	Single	33	33	0.75 (0.42, 1.32)
	Divorced	4	0	–
	Widowed	0	1	–
Work experience	≤5 years	112	94	1
	>5 years	22	18	1.02 (0.52, 2.02)
Working days	≤5 days per week	8	6	1
	>5 days per week	103	129	1.41 (0.55, 4.88)
Monthly salary (Eth. birr)	≤3,200	115	91	1.00
	>3,200	19	21	0.71 (0.36, 1.41)
PPE use on duty	No	9	6	1.00
	Yes	25	25	0.86 (0.49, 1.53)
Training on Health and safety before employment	No	89	61	1.00
	Yes	45	51	0.60 (1.05, 2.02)*
On job training	No	65	52	1.00
	Yes	69	60	0.92 (0.56, 1.52)
Work related violence	No	98	89	1.00
	Yes	36	23	1.48 (0.81, 2.71)*
Smoking cigarette	No	86	64	1.00
	Yes	48	48	0.73 (0.44, 1.22)
Drinking alcohol	No	123	102	1.00
	Yes	11	10	0.91 (0.37, 2.21)
Chewing chat	No	68	54	1.00
	Yes	66	58	0.90 (0.54, 1.49)
Sleeping problem	No	47	70	1.00
	Yes	88	41	3.10 (1.83, 5.20)*
Job satisfaction	No	25	10	1.00
	Yes	109	102	0.43 (0.19, 0.94)*
Employment condition	Permanent	71	69	1
	Temporary/contract	63	43	1.46 (0.87, 2.43)*

* Variables which were significant (*p*-value < 0.25) in bi-variate analysis and became a candidate in multivariate analysis.

#### 3.5.2 Multivariable logistic regression analysis

The study found a significant association between health and safety training before employment, sleeping problems, and employment conditions and occupational injuries. Training on health and safety measures before employment significantly reduced the risk of occupational injuries in 57% of workers [AOR: 0.43, 95% CI (0.24, 0.80)]. Workers with sleeping problems were 3.28 times more likely to experience an occupational injury [AOR: 3.28, 95% CI (1.86, 5.78)] than those without sleeping problems. Temporary workers were 2.14 times more likely to face occupational injury than permanent workers who engage in waste collection [AOR: 2.14, 95% CI (1.16, 3.95)] (Table 6).

**Table 6:** Multi-variable logistic regression of occupational injury with predictor variables among solid waste collectors in Jigjiga city, eastern Ethiopia, June 25th to July 25th 2023GC (*n* = 246).

Variable	Predictor	Occupational injuries in the past 12 months (246)		COR (95% CI)	AOR (95% CI)
		Yes	No		
Employment Condition	*Permanent*	71	69	1.00	1.00
	*Temporary*	63	42	1.46 (0.87, 2.43)	2.14 (1.16, 3.95)*
Job satisfaction	*No*	25	10	1.00	1.00
	*Yes*	109	101	0.43 (0.19, 0.94)	0.43 (0.18, 1.01)
Sleeping problem	*No*	47	69	1.00	1.00
	*Yes*	87	41	3.11 (1.84, 5.26)	3.28 (1.86, 5.78)*
Training on Health and safety before employment	*No*	89	61	1.00	1.00
	*Yes*	45	51	0.60 (1.05, 2.02)	0.43 (0.24, 0.80)*
Work related violence	*No*	98	89	1.00	1.00
	*Yes*	36	22	1.48 (0.81, 2.71)	1.59 (0.80, 3.20)

* Significant with *P* < 0.05. CI, confidence interval; COR, crude odds ratio; AOR, adjusted odds ratio.

### 3.6 Observational findings

During data collection, no one has worn overall clothing while on duty; only 26.12% of the waste collectors were observed using one or more PPE. On utilization observed during data collection, 20 (30.77%), 45 (69.23%), 24 (36.92%) and 41 (63.07%) were new, water proof, well dressed and perforated respectively. During the observation, no one has worn overall clothing. While on duty, only 2 (3.08%) workers wore boots, while the others wore short shoes and sleepers, which could be the source of occupational injuries. At the time of observation, there were no carts present. The condition of waste collection sacks was punctured, overfilled, and some were difficult to lift, which could result in injuries during the collection and lifting of solid wastes.

## 4 Discussion

The prevalence of occupational injuries was found to be 54.7% (95% CI: 48.2%, 60.6%). Factors that were significantly associated with occupational injuries were training on health and safety before employment, sleeping problems, and employment conditions. The overall prevalence of occupational injuries in this study is similar to studies done in Kigali, Rwanda, at 51.5% (John et al., 2018). Another study undertaken in Diredawa, Ethiopia, shows the same prevalence as my study, which is 53.2% (23). However, the prevalence observed in this study is higher than studies done in Tanzania at 40.9% (17) and Addis Ababa, Ethiopia, at 43.7% (21), but lower than studies done in Harar at 60.4% (20) and Gondar and Bahir dar towns in Ethiopia at 63.9% (22). This difference might be due to variations in regulations and cultures among the residents.

This study has depicted that the most common injured body parts were 26.49% hand injuries, followed by finger injuries, legs injuries, and knee injuries. This figure of 22.7% is markedly greater in Gonder and Bahirdar towns in northern Ethiopia and relatively lower in Harar Ethiopia, which is 56.8% (20, 22). This is due to the fact that manually loading, unloading, and picking up waste without wearing appropriate gloves and shoes can increase the probability of a cut, bruises, and ruptures.

On the other hand, the study also found that the most common types of occupational injuries among solid waste collectors were 36% abrasion/laceration, 24% burn, 18% cut, and 8% puncture. Similar studies show that the rate of abrasion/laceration and cut are the most reported types of injuries among solid waste collectors, according to studies done in Ethiopia and Tanzania (17, 23, 25, 26). This could be because the majority of solid waste collectors collect waste by hand, and workers continue to handle a wide variety of objects and containers (sacks) of varying size and weight without wearing appropriate protective equipment.

In this study, it is illustrated that the majority of solid waste collectors did not receive training on health and safety measures before employment. The incident of occupational injuries was associated with health and safety training as the result of the study reveals that training on health and safety measures before employment was more likely to be protective 57% to experience an occupational injury [AOR: 0.43, 95% CI (0.24, 0.80)] than workers who did not receive training on health and safety measures before employment. This findings was supported by another studies conducted in Brazil, Ethiopia and Egypt there were statistically significant differences among waste collectors who received training programs before and after intervention (22, 23, 27, 28).

The result of the study has revealed that the occurrence of any type of occupational injuries is significantly related with sleeping problems. Those workers who experience sleeping problems were 3.28 times more likely to experience an occupational injury [AOR: 3.28, 95% CI (1.86, 5.78)] than those without sleeping problems. This finding is similar with other studies conducted in a systematic review and in Ethiopia (21, 22, 29). This could explain that sleeping problems affect the ability to maintain wakefulness, concentration, ability in assessing or watching the work environment and working conditions.

Temporary workers were 2.14 times more likely to face occupational injury than permanent workers who engage in waste collection [AOR: 2.14, 95% CI (1.16, 3.95)]. A study done in India explains the six-fold difference in daily pay between regular and casual employees. Regular employees received medical benefits; however, casual workers employed by private contractors or under contract were not eligible. Only permanent waste collectors were discovered to have access to medical facilities (30). This indicates that the temporary workers do not get benefits and other workers’ rights and face many challenges and health problems for which no one gives a helping hand.

## 5 Strengths and limitations

The study utilized common questionnaires from previous research and International labour organizations (ILO) materials, which were updated based on outcome factors and observations. This approach made it easier to compare occupational injuries from a national and global perspective, enhancing the study's strength. The study's limitations include not considering the health issues of solid waste collectors and potential recall bias. The interview procedure and employees’ reluctance to discuss past injuries may have contributed to bias. High turnover rates may not have reported accidents. The cross-sectional design makes it difficult to determine cause and effect, and self-reported injuries may have been over- or under-reported.

## 6 Conclusion

The results of this study have shown higher prevalence rate of occupational injury. It identified that Training on Health and safety before employment, sleeping problem and Employment condition was the interpreter factors associated with occupational injury. Based on the above findings and conclusions for sustained prevention and control of occupational injuries, the following recommendations have been suggested to different stakeholders: Jigjiga City Administration Sanitation and Beautification Office and Somali Region Urban Development and Construction Bureau, Bureau of Labor and social affairs, MSEs (cooperatives), the community, and researchers. The study suggest provides suitable and complete protective equipment's regularly, such as rubber or heavy-duty gloves, face masks, boots, and overalls. Also, regular occupational health and safety training should be provided to solid waste collectors in Jigjiga municipality to educate them about hazards and injuries, and to ensure they get enough sleep. Workers should also be educated on their duty to avoid conflicts and rule violations during working hours. Waste collection companies or unions should consider regular equipment supply to improve compliance and use mechanical techniques in their daily work.

## Data Availability

The original contributions presented in the study are included in the article/Supplementary Material, further inquiries can be directed to the corresponding author.
