# Supplementary material for: Prevalence of occupational injuries and associated factors among solid waste collectors in Jigjiga city, eastern Ethiopia: a cross-sectional study design
Source: Front Epidemiol. 2024 Nov 12;4:1439038. doi: 10.3389/fepid.2024.1439038 (PMC11588437; doi:10.3389/fepid.2024.1439038)
Supplement: Supplementary file 1 [file Table1.docx]

Supplementary file

Jigjiga City Administration Sanitation & Beautification (JCA B&S) Office & MSEs

N=247

JCA B&S Office

(Sample size: 100)

MSEs

(Sample size: 147)

**Total Sample Size 247**

JCA B&S Office

(N_1_=100)

MSEs

(N_2=_147)

Figure 1: Schematic presentation of the sampling procedure for the selection of sample Solid Waste Collectors at JCA Sanitation & Beautification Office & MSEs in Jigjiga City, Somali Region, Ethiopia.
